# Evaluating the Efficacy of Electronic Screening, Brief Intervention, and Referral to Treatment (e‐SBIRT) for Gambling: An Online Pilot Randomised Trial

**DOI:** 10.1002/jclp.70188

**Published:** 2026-07-10

**Authors:** Simon Wright, Martyn Quigley, Simon Dymond

**Affiliations:** ^1^ School of Psychology Swansea University Swansea UK; ^2^ Department of Psychology Reykjavík University Reykjavík Iceland

**Keywords:** brief intervention, e‐SBIRT, gambling, motivational interviewing, randomised pilot trial

## Abstract

**Objectives:**

To investigate the efficacy of electronic screening, brief intervention, and referral to treatment (e‐SBIRT) at improving gambling outcomes and increasing help‐seeking.

**Methods:**

We conducted a two‐arm, randomised online pilot trial (*n* = 83) comparing an e‐SBIRT intervention with an active control over 12 weeks. The brief intervention was informed by motivational interviewing and incorporated personalised normative feedback, information provision, and relapse‐prevention components. Eligible participants were aged 18 or older, resided in the UK and had scores indicating at least moderate severity gambling.

**Results:**

Participants (54 [65.1%] male; mean [SD] age = 40.58 [12.75] years, mean [SD] PGSI = 7.16 [5.58]) in the e‐SBIRT and control conditions showed improvements in gambling harms (*p* = 0.033) and perceived ability to control gambling (*p* = 0.029). No significant effects of condition assignment or condition x time interactions were observed. However, exploratory analyses of individual model coefficients suggested greater improvement in perceived ability to control gambling among participants receiving e‐SBIRT at 12 weeks (*p* = 0.043). Exploratory analyses also suggested higher rates of help‐seeking at 12‐week follow‐up among participants receiving e‐SBIRT.

**Conclusion:**

Overall, e‐SBIRT did not demonstrate clear advantages over assessment and information provision alone. Further research should prioritise refining intervention components and evaluating SBIRT approaches in settings that better reflect its opportunistic delivery model.

## 1 Introduction

Gambling disorder (GD) is characterized by a persistent pattern of gambling despite significant distress or impairment (American Psychiatric Association 2013). Gambling is associated with a broad spectrum of harms, including adverse effects on financial stability, physical and psychological health, relationships, employment, and education (Abbott 2020; Public Health England 2021; Wardle et al. 2019). In Great Britain, the prevalence of “problematic gambling”—a term commonly used to describe those who score 8 or higher on the *Problem Gambling Severity Index* (PGSI; Ferris and Wynne 2001), has been estimated at 2.5% (Gambling Commission 2024). Despite this, help‐seeking rates remain low, both internationally (Bijker et al. 2022) and in the UK, (GambleAware 2024; Office for Health Improvement and Disparities 2024) highlighting the importance of interventions that can effectively motivate change.

One approach that has been recommended to support behaviour change and engagement with treatment is motivational interviewing (Forman et al. 2025; Yakovenko et al. 2015). Motivational interviewing (MI) was developed as a method to help people work through ambivalence and commit to change (Miller 1983). Rooted in client‐centred therapy, MI combines supportive and empathic counselling (Rogers 1959) with consciously directed action towards resolving ambivalence in the direction of change. In practice, MI is typically brief and delivered in one or two sessions and can be provided as a standalone intervention or combined with other methods. In the UK, the current guidelines for the identification, assessment and management of gambling‐related harms recommend that MI be considered to strengthen people's confidence and commitment to change, or to encourage people who express reservations about entering treatment (National Institute for Health and Care Excellence 2025).

Evidence for the effectiveness of MI in the treatment of gambling problems is mixed. Yakovenko et al. (2015) conducted a systematic review and meta‐analysis of MI for GD. They found that, at posttreatment, participants receiving MI reported significantly lower gambling frequency and expenditure compared to those receiving no treatment or non‐MI interventions. At follow‐up (up to 1 year), MI remained associated with reduced gambling frequency, although differences in gambling expenditure were no longer significant. In contrast, Forman et al. (2025) reported little evidence of benefit. Their meta‐analysis of MI‐informed interventions showed outcomes that were largely equivalent to non‐MI control conditions at posttreatment and follow‐up. Forman et al. (2025) noted that this discrepancy may stem from different inclusion criteria: their review examined MI as a standalone intervention, whereas Yakovenko et al. (2015) included some studies that combined MI with cognitive behavioural components. However, despite mixed evidence regarding the effectiveness of MI for gambling problems, help‐seeking rates remain low (Bijker et al. 2022; GambleAware 2024; Office for Health Improvement and Disparities 2024), underscoring the need for interventions that can reduce gambling‐related harm as well as increase help‐seeking behaviour.

One approach which is capable of reducing harm and promoting uptake of support is screening, brief intervention and referral to treatment (SBIRT; Babor et al. 2007). SBIRT is designed to quickly assess the severity of harm from addictive behaviours, provide personalised feedback and education on risk, and then deliver a brief intervention (often based on MI) with referral to treatment as needed (Babor et al. 2007). Typically, SBIRT interventions are delivered opportunistically in clinical settings, to identify and intervene with individuals who may not otherwise seek treatment. SBIRT programmes have demonstrated effectiveness in improving alcohol‐related outcomes, including reductions in use, related consequences, and repeat emergency department visits (Barata et al. 2017). More recently, SBIRT approaches have been adapted for application to gambling. Heinlein et al. (2022) found that a single‐arm pilot feasibility study of a gambling‐specific SBIRT (*n* = 15) led to reductions in the number of days gambled and the median amount of money spent at 1‐month follow‐up. With advances in technology, client‐accessed electronic SBIRT (e‐SBIRT) programmes have become increasingly common. In a systematic review and meta‐analysis, Jones et al. (2024) concluded that e‐SBIRT may offer benefits comparable to in‐person delivery while being less resource‐intensive in terms of cost and time. In a recent study, Wright et al. (2025) examined the acceptability of core e‐SBIRT components for gambling using a mixed‐methods design. Participants with moderate to high severity gambling were recruited online, engaged with the e‐SBIRT components once, and subsequently completed measures of acceptability. Findings indicated that participants perceived the e‐SBIRT components as helpful and impactful in relation to their gambling and reported an increased likelihood of seeking help following engagement. Consistent with these findings, qualitative feedback further supported the acceptability of the intervention.

While the acceptability of e‐SBIRT components for gambling has been indicated, their efficacy has not yet been examined. To address this gap, the present study aimed to provide a preliminary evaluation of the efficacy of core e‐SBIRT components, where the brief intervention was informed by MI. It was hypothesized that participation in e‐SBIRT components would lead to improvements in gambling harms, frequency, expenditure, symptom severity, perceived ability to control gambling and help‐seeking, relative to an active control condition at 4‐ and 12‐week follow‐up assessments (see OSF preregistration for full hypotheses: https://osf.io/cq5j9). As a pilot study, findings are intended to offer initial evidence regarding the efficacy of e‐SBIRT components in an online context rather than opportunistic clinical settings. In doing so, this study contributes to the emerging evidence base of MI‐informed digital interventions for gambling harm and helps clarify the potential role of e‐SBIRT within future pathways of support.

## 2 Methods

### 2.1 Design

This study was a two‐arm randomised online pilot trial. Data were collected at baseline, 4 and 12‐weeks postintervention. Reporting of this study adheres to the APA's Journal Article Reporting Standards (JARS) for quantitative research designs where applicable.

### 2.2 Protocol Registration

The study protocol was preregistered on the Open Science Framework (https://osf.io/cq5j9). One deviation from the preregistered protocol occurred: a prespecified primary outcome was removed from the analyses because the measure was administered outside its validated timeframe.

### 2.3 Participants and Procedure

Recruitment took place using the Prolific website. Participants were contacted using Prolific IDs and invited to take part in a ‘multi‐part study looking at a tool to reduce harm from gambling’. Participants were eligible if they were aged 18 or older, resided in the UK and scored ≥ 3 on the PGSI (indicating at least moderate risk of problem gambling; Ferris and Wynne 2001). This threshold was chosen to align with the preventive and early‐intervention focus of e‐SBIRT, which aims to engage individuals experiencing harm who may not yet seek formal treatment (Babor et al. 2007). During the consent stage, participants were informed not everyone would receive the same intervention, and after agreeing to take part, participants were randomised to the intervention or control arm. Randomisation occurred in a 1:1 ratio and was carried out using the Qualtrics Randomiser algorithm programmed into the survey platform. Once allocated to conditions, participants supplied demographic information (age, gender, education, employment and socio‐economic status) and were asked to provide a brief treatment history. Participants then filled in the outcome measures and completed the allocated intervention. Two additional surveys were sent after 4 and 12 weeks with a £2 payment for each. Recruitment took place between January and May 2025.

### 2.4 Primary Outcome Measures

#### 2.4.1 Quantitative

*Gambling harms*: Reported harms from one's own gambling was assessed using the Short Gambling Harm Screen (SGHS; Browne et al. 2018), a 10‐item measure assessing the presence and degree of harm caused by gambling. Although the SGHS is typically administered over a 12‐month timeframe, we opted to use a past‐month timeframe to capture proximal harms and recent experiences that may be responsive to brief intervention over short follow‐up periods. Items are endorsed in a binary (*yes/no*) format in response to a prompt (e.g., ‘*In the past month, have you experienced any of the following due to your gambling: Felt ashamed of my gambling’*). Total scores range from 0 to 10, with higher scores indicating greater gambling harm. Internal consistency of the SGHS at baseline in the present sample was good (Cronbach's *α* = 0.85).

*Gambling frequency*: Gambling frequency was assessed by asking participants, via open text response, “*In the past month, how many hours do you think you gambled*?”

*Gambling expenditure*: Gambling expenditure was assessed by asking participants, via open text response, “*In the past month, how much money (£) did you spend on gambling?*”

*Gambling symptom severity*: Gambling symptom severity was assessed using the Gambling Symptom Assessment Scale (G‐SAS; Suck Won Kim et al. 2009), a 12‐item measure of gambling symptom severity. The G‐SAS assesses symptoms over the past week (e.g., “*During the past week, how many times did you experience urges to gamble?*”) with items scored on a 0 to 4 scale using item‐specific response options. Total scores range from 0 to 48, with higher scores indicating greater symptom severity. G‐SAS scores are categorised as mild (8–20), moderate (21–30), severe (31–40), or extreme (41–48). Internal consistency at baseline in the present sample was excellent (Cronbach's *α* = 0.92).

*Perceived ability to control gambling:* Perceived ability to control gambling was assessed using the single item “*How confident are you that you can limit or stop your gambling*?” Response options ranged from 0 (*not at all*) to 10 (*very confident*).

### 2.5 Qualitative

*Past month help‐seeking:* To assess help‐seeking behaviour, participants were asked “*Have you sought support for your gambling in the past month*?” Response options were binary (*yes*/*no*). If participants responded affirmatively, they were asked “*Please describe the treatment you sought*.” via an open text box.

### 2.6 Secondary Outcomes

*Quality of life:* Quality of life was measured using the EUROHIS—Quality of Life scale (Schmidt et al. 2006), an 8‐item index used to assess overall quality of life (e.g., ‘*How would you rate your quality of life*?’). Response options range from 1 (*very poor*) to 5 (*very good*), with higher scores indicating higher quality of life.

### 2.7 Interventions

#### 2.7.1 e‐SBIRT Components

**Figure 1:**
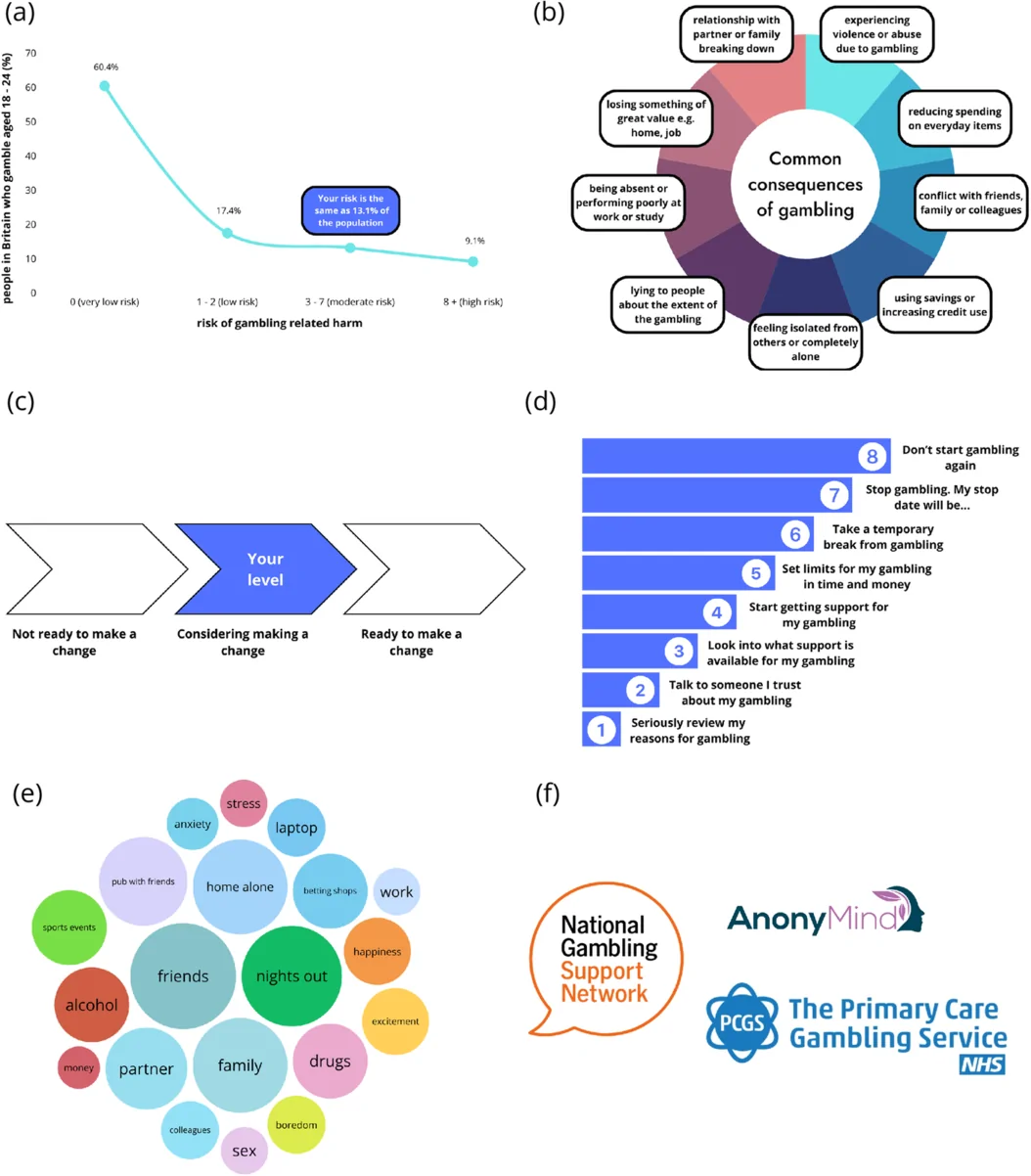
Example screenshots of the e‐SBIRT intervention; (a) Personalized normative feedback based on age and gambling severity, (b) Information provision on the consequences of gambling, (c) Readiness to change assessment with feedback, (d) Goal setting ladder, (e) Relapse prevention and (f) Signposting page detailing support agencies.

The design of the e‐SBIRT components was guided by Jones et al. (2024). The brief intervention was informed by MI (Miller and Rollnick 2002) and was further shaped by Keshani et al. (2025) and current UK gambling‐related harms guidelines (National Institute for Health and Care Excellence 2025). Example screenshots are displayed in Figure 1, and an anonymous copy can be accessed at https://tinyurl.com/y2pe7e6s.

*Screening*: Participants were screened using the PGSI (Ferris and Wynne 2001). The PGSI assesses gambling severity and contains 9 items (e.g., ‘*Thinking about the past 12 months… Have you bet more than you could really afford to lose?’*) rated on a 4‐point scale (0 ‘*never*’ to 3 ‘*almost always*’). Participant's scores can be placed into four categories: non‐problem (score of 0), low risk (score of 1–2), moderate risk (score of 3–7) and high risk of “problematic gambling” (score of 8 or higher).

*Brief intervention*: After completion of the PGSI, participants were given personalised normative feedback (PNF) based on their age and gambling severity score (e.g., ‘*We compared this with other people who gamble in your age group [18–24], and found that you scored higher than 90.9% of people*’). Respondents then viewed information on the consequences of gambling (e.g., ‘*A recent survey found that 4 in 10 people who engage in high risk gambling experienced at least one ‘severe’ consequence due to their gambling*’) and completed a readiness to change assessment with feedback (e.g., ‘*Your answers suggest that you are considering making a change to your gambling right now*’). Depending on responses, users then completed a decisional balance exercise (e.g., ‘*Use the exercise below to rank your reasons for gambling in terms of how important they are to you*’) and then exercises in goal setting (e.g., ‘*In this exercise you are going to set some goals*’) and relapse prevention (e.g., ‘*Now it's time to create a plan for how to cope with these triggers without gambling*’).

*Referral to treatment*: After completion of the brief intervention, participants viewed a signposting page encouraging them to seek support from a range of agencies. The listed agencies included the National Gambling Support Network, the Primary Care Gambling Service and the NHS website for gambling addiction.

### 2.8 Control

The control condition was designed as an active comparator to control for nonspecific intervention effects, such as exposure to gambling‐related information and study engagement. Specifically, participants completed the PGSI, received feedback on their gambling severity category, viewed information on the consequences of gambling, safety tips to prevent harm and the same signposting page as those in the intervention arm. Control participants did not receive the core e‐SBIRT components designed to drive behaviour change, including PNF, decisional balance, goal setting, or relapse‐prevention content. Upon completion of the study, participants in the control arm were given access to the e‐SBIRT components via the study debrief form.

### 2.9 Analysis

Analyses were conducted using SPSS (version 30.0.0) and R (version 4.5.1; *lme4* package). Descriptive statistics were reported for all outcomes, either as unweighted frequencies and percentages or as means and standard deviations (SDs). To evaluate the impact of the intervention, linear mixed‐effects models were fitted separately for each quantitative primary outcome. Each model included a random intercept for participants to account for between‐person variability in baseline outcome levels. Random slopes for time were not specified; therefore, the models estimate average change over time rather than individual differences in change trajectories. Baseline observations were included as the initial time point (time modelled categorically), rather than entered as a separate baseline covariate. Five separate models examined the fixed effects of time, condition, and the time x condition interaction. Effect sizes are presented as fixed‐effect estimates (β) with 95% confidence intervals. Models were estimated using restricted maximum likelihood (REML), allowing inclusion of all available data under the assumption that data were missing at random. Post hoc pairwise comparisons were conducted using the *emmeans* package in R, with Bonferroni correction. An alpha level of 0.05 was adopted for all analyses.

## 3 Results

### 3.1 Sample and Demographics

**Figure 2:**
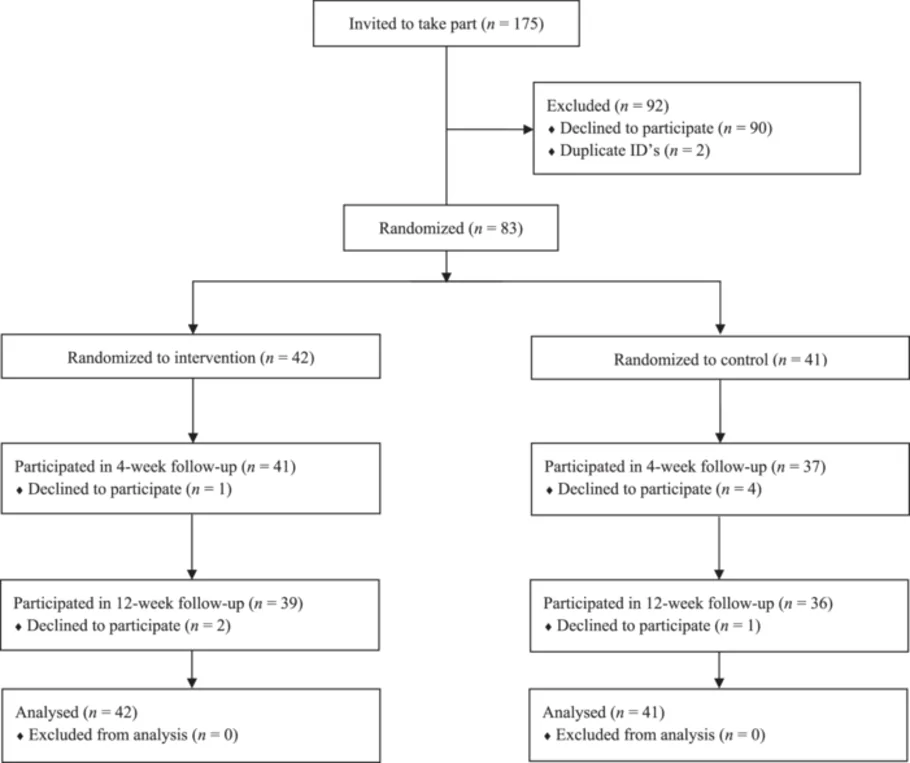
Flow of participants through each stage of the study.

A total of 83 (intervention *n* = 42, control *n* = 41) participants completed the baseline survey (see Figure 2). Demographic characteristics are presented in Table 1. The mean age was 40.58 (SD = 12.75), 54 were male (65.10%), 63 (75.90%) reported their ethnicity as White and 42 (50.60%) held a university degree or higher. Most participants were in full time employment (*n* = 59; 71.08%) and 61 participants (73.49%) reported a past year income of over £25,000. There were no significant differences between the intervention and control groups for any demographic characteristic at baseline (*p* values > 0.05).

**Table 1:** Demographics characteristics of the sample at baseline (*N* = 83).

Demographic characteristic^a^	Intervention (*n* = 42)	Control (*n* = 41)	Statistic	*p*
**Age, mean (SD) y**	38.88 (12.47)	42.32 (12.94)	*t*(81) = − 1.23	*p* = 0.22
**Gender**				
Male	29 (69.0)	25 (61.0)	*χ* ^2^(1) = 0.60	*p* = 0.44
Female	13 (31.0)	16 (39.0)		
**Ethnicity**				
White	34 (81.0)	29 (70.7)	*χ* ^2^(1) = 1.19	*p* = 0.28
Other	8 (19.0)	12 (29.3)		
**Education**				
No university degree	25 (59.5)	16 (39.0)	*χ* ^2^(1) = 3.49	*p* = 0.062
University degree or higher	17 (40.5)	25 (61.0)		
**Employment**				
Full‐time employed	32 (76.2)	27 (65.9)	*χ* ^2^(1) = 1.08	*p* = 0.30
Other	10 (23.8)	14 (34.1)		
**Income**				
Lower income (< £25,000)	12 (28.6)	10 (24.4)	*χ* ^2^(1) = 0.19	*p* = 0.67
Higher income (≥ £25,000)	30 (71.4)	31 (75.6)		

^a^ Data are presented as number (percentage) of study participants unless otherwise indicated.

In terms of gambling outcomes, the sample reported an average of 4.01 harms (SD = 3.00), an average past‐month frequency of 17.77 h (SD = 28.07), an average past‐month expenditure of £176.55 (SD = 164.52) and an average perceived ability to control gambling of 6.78 (SD = 2.32). For past‐week gambling symptom severity, the sample reported average G‐SAS scores of 17.88 (SD = 8.25), indicating mild gambling symptoms. At baseline, the sample reported PGSI scores indicating moderate risk gambling (M = 7.16, SD = 5.58). There were no significant differences between the intervention and control groups for any outcome of interest at baseline (*p* values > 0.05; see Table 2).

**Table 2:** Baseline scores for quantitative outcomes of interest for the intervention and control groups.

Outcome of interest	Intervention (*n* = 42)	Control (*n* = 41)	Statistic	*p*
Gambling harms, mean (SD)	4.21 (3.06)	3.80 (2.96)	*t*(81) = 0.62	*p* = 0.54
Gambling frequency, median (IQR)	10.00 (15.25)	10.00 (12.50)	*U* = 834.5	*p* = *.81*
Gambling expenditure, median (IQR)	145.00 (150.00)	100.00 (180.00)	*U* = 819.5	*p* = 0.70
Gambling symptom severity, mean (SD)	17.81 (8.39)	17.95 (8.21)	*t*(81) = − 0.08	*p* = 0.94
Perceived ability to control gambling, median (IQR)	6.50 (5.00)	7.00 (2.50)	*U* = 754.5	*p* = 0.33

### 3.2 Gambling Outcomes—Linear Mixed‐Effects Models

In terms of the primary outcomes, five separate linear‐mixed effects models were conducted to examine the effect of the e‐SBIRT components on outcome measures compared to the control condition over time.

There were no significant main effects of condition assignment on any outcome (all *p*s > 0.05). However, significant main effects of time were observed for gambling harms, *F*(2. 151.13) = 4.13, *p* = 0.018, and perceived ability to control gambling, *F*(2. 151.26) = 3.48, *p* = 0.033. No main effects of time were found for gambling frequency, expenditure or symptom severity (all *p*s > 0.05). In addition, no main interaction effects were observed for any outcome (all *p*s > 0.05).

**Figure 3:**
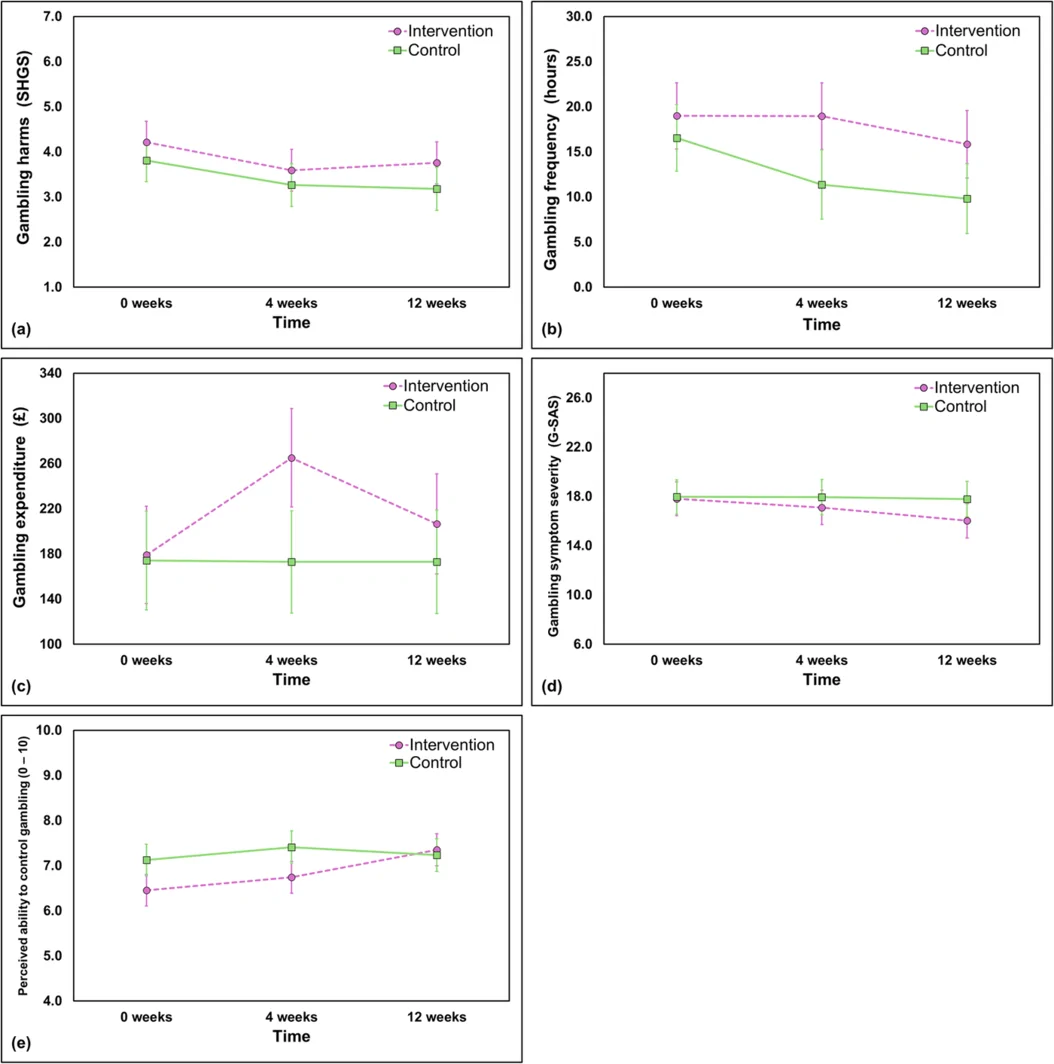
Plots of the estimated marginal means with standard error bars across time for each condition: (a) gambling harms, (b) gambling frequency, (c) gambling expenditure, (d) gambling symptom severity and (e) perceived ability to control gambling.

The estimates of the fixed effects for each model are displayed in Table 3, and Figure 3 displays the estimated marginal means across time for each condition.

**Table 3:** Estimates of fixed effects of time, condition, and time x condition interaction on primary outcome measures.

	Gambling harms (SGHS)			Gambling frequency (hours)		
Predictors	Estimate	*t*	*p*	Estimate	*t*	*p*
Intercept	4.21	9.14	< 0.001***	18.98	5.18	< 0.001***
Time (Ref: Week 0)						
Week 4	−0.62	−2.0	0.0480*	−0.035	−0.011	0.992
Week 12	−0.46	−1.44	0.151	−3.14	−0.92	0.360
Condition (Ref: Intervention)						
Control	−0.41	−0.62	0.534	−2.44	−0.47	0.640
**Interaction**						
Control, Week 4	0.080	0.18	0.861	−5.14	−1.06	0.292
Control, Week 12	−0.17	−0.37	0.715	−3.59	−0.73	0.470

	Gambling expenditure (£)			Gambling symptom severity (G‐SAS)			
Predictors	Estimate	*t*	*p*	Estimate	*t*	*p*	
Intercept	178.91	4.15	< 0.001***	17.81	12.92	< 0.001***	
Time (Ref: Week 0)							
Week 4	86.12	1.99	0.0489*	−0.71	−0.70	0.488	
Week 12	27.61	0.63	0.532	−1.79	−1.72	0.087	
Condition (Ref: Intervention)							
Control	−4.76	−0.078	0.938	0.14	0.072	0.943	
**Interaction**							
Control, Week 4	−7.40	−1.40	0.165	0.70	0.47	0.639	
Control, Week 12	−2.12	−0.033	0.973	1.62	1.08	0.281	

	Perceived ability to control gambling (0–10)		
Predictors	Estimate	*t*	*p*
Intercept	6.45	18.58	< 0.001***
Time (Ref: Week 0)			
Week 4	0.29	1.10	0.275
Week 12	0.90	3.36	< 0.001***
Condition (Ref: Intervention)			
Control	0.67	1.36	0.178
**Interaction**			
Control, Week 4	−0.00022	−0.001	1.000
Control, Week 12	−0.79	−2.040	0.0430*

*Note:* *p < 0.05. ***p < 0.001.

Abbreviation: REF, reference category.

The fixed effects analysis revealed a significant decrease in gambling harm scores at 4 weeks (*β* = −0.62, 95% CI [ − 1.23, −0.01], *p* = 0.048). Post hoc pairwise comparisons using estimated marginal means confirmed a significant reduction in gambling harms from baseline to 4 weeks (M difference = 0.58, *p* = 0.033). There were no significant differences between baseline and 12 weeks (*p* = 0.058) or between 4 and 12 weeks (*p* = 1.00).

For perceived ability to control gambling, the fixed effects estimates demonstrated a significant increase at 12 weeks relative to baseline (*β* = 0.90, 95% CI [0.36, 1.42], *p* < 0.001). The omnibus condition x time interaction was not statistically significant, *F*(2, 151.26) = 2.71, *p* = 0.070, indicating that overall trajectories did not differ significantly between conditions. However, examination of the individual model coefficients revealed a significant interaction estimate at Week 12 (*β* = −0.79, 95% CI [ − 1.55, −0.02], *p* = 0.043), suggesting that participants in the intervention condition reported greater improvements in perceived ability to control gambling than those in the control condition at this time point. Given the absence of a significant omnibus interaction effect, this finding should be considered exploratory and interpreted with caution. Post hoc pairwise comparisons indicated a significant increase in perceived ability to control gambling from baseline to 12 weeks (*M* difference = −0.51, *p* = 0.029), whereas differences between baseline and 4 weeks (*p* = 0.397) and between 4 and 12 weeks (*p* = 0.798) were not significant.

In terms of secondary outcomes, there was no effect of time or condition assignment on participant quality of life scores (Material S1).

### 3.3 Past Month Help‐Seeking

At baseline, 4/83 participants indicated they had sought help for their gambling in the past month (*n* = *2* intervention, *n* = *2* control). At 4‐week follow‐up, this had reduced to three (*n* = *2* intervention, *n* = *1* control) and at 12 weeks, this had increased to seven (*n* = *6* intervention, *n* = *1* control). The 14 affirmative responses were made by 12 different participants (intervention: *n* = *9*, control: *n* = *3*). Exploratory analysis confirmed a trend toward higher rates of help‐seeking among participants in the intervention group compared to the control group (21.4% vs. 7.3%), *χ* ^2^(1, *n* = 83) = 3.34, *p* = 0.068. Of the nine participants who described the help they sought, three engaged in formal treatment, three accessed online resources, two discussed their gambling with peers, and one enrolled in self‐exclusion software.

## 4 Discussion

This pilot trial was the first to evaluate the efficacy of core e‐SBIRT components, where the brief intervention was informed by MI. Across both conditions, participants showed reductions in gambling harms and increased perceived ability to control gambling. Contrary to our hypotheses, no main effect of condition or condition x time interaction was identified. However, exploratory examination of the model coefficients suggested a potential advantage for participants receiving the e‐SBIRT components in perceived ability to control gambling at 12 weeks. Exploratory analyses also indicated higher levels of help‐seeking among participants receiving e‐SBIRT at follow‐up.

Although participants showed improvements over time, the e‐SBIRT components did not produce benefits beyond those observed in the control condition. This finding is consistent with Forman et al. (2025), who reported that outcomes from MI‐informed interventions were largely equivalent to non‐MI control conditions at post‐treatment. The largely null findings observed in the present study are also consistent with the broader literature on PNF interventions for gambling, which has shown limited effects (Peter et al. 2019; Saxton et al. 2021). One possible explanation is that the inclusion of multiple intervention components resulted in theoretical inconsistency or component interference, whereby some elements dilute or undermine the intended mechanisms of change (Saxton et al. 2021). In the present study, our intervention combined MI‐informed elements with PNF, information provision, and relapse‐prevention content, which may have limited the effectiveness of MI‐consistent processes. To understand this further, future research on brief interventions may benefit from greater coherence with MI principles or the inclusion of a pure MI comparator group.

Another explanation for the lack of between‐group differences is that the control intervention may have promoted the changes in outcome variables. Participants in both groups demonstrated improvements in gambling harms and perceived ability to control gambling, and both conditions included an information provision component. Notably, Keshani et al. (2025) identified information provision as the fourth most effective change technique for gambling treatment (out of 19 techniques), with clinical consensus supporting its effectiveness. This component alone may therefore have been sufficient to elicit improvements across both groups. The lack of differential effects between conditions may also reflect the context in which the intervention was delivered. Unlike traditional SBIRT models delivered opportunistically during clinical encounters (Babor et al. 2007; Barata et al. 2017), our intervention was delivered online, where readiness to act on screening feedback may be lower. Future evaluations should therefore examine SBIRT delivered in contexts more closely aligned with its’ opportunistic delivery model (e.g., primary care health services and/or immediately following gambling episodes).

In this study, 12 participants (14.4%) sought help for their gambling across both conditions. Globally, only 1 in 25 (4%) people with moderate‐risk PGSI scores report seeking help (Bijker et al. 2022). Given our sample largely comprised individuals with moderate PGSI scores, the help‐seeking rate observed here was notably higher. One possible explanation is that participants in the intervention arm felt more confident about seeking support. Although no significant condition x time interaction was observed for perceived ability to control gambling, exploratory examination of the model coefficients suggested a potential advantage for the intervention group at 12 weeks. Furthermore, 75% (9 of 12) of participants who sought help were in the intervention condition. Taken together, these findings may indicate a possible relationship between perceived ability to control gambling and help‐seeking behaviour. However, given the exploratory nature of the analyses, the non‐significant omnibus interaction test, and the small number of help‐seeking events observed, these findings should be interpreted cautiously. Notwithstanding, the present findings highlight the potential of e‐SBIRT to address an important gap in existing gambling interventions. Furthermore, the proportion of participants reporting help‐seeking in the present study may represent an underestimate. We relied on a single‐item measure, whereas Rodda et al. (2018) demonstrated that the use of a more comprehensive help‐seeking instrument can substantially increase reported rates. In their study, 277 participants with scores indicating high risk gambling completed both a one‐item and an expanded 14‐item help‐seeking questionnaire, where the latter identified substantially higher lifetime professional help‐seeking (70%) than the single‐item measure (22%). Future research should therefore use more comprehensive instruments to better capture the potential of brief interventions to encourage help‐seeking.

Our findings should be interpreted in light of certain limitations. Despite several outcomes descriptively favouring the intervention condition (e.g., gambling symptom severity, perceived ability to control gambling), no main effect of condition assignment was detected. One explanation may be that large standard errors across outcomes reduced the precision of estimates, limiting our ability to detect between‐group differences. To overcome this, future studies which use linear‐mixed effects models should use simulation‐based power analysis. In addition, our models included random intercepts but did not incorporate random slopes for time. As a result, the analyses estimate average trajectories of change and do not model potential heterogeneity in individual change over time. Furthermore, although improvements were observed over time across both conditions, these changes were largely parallel and may reflect regression to the mean, assessment effects, or natural change rather than intervention‐specific effects. In the absence of a no‐treatment control group, time effects should not be interpreted as evidence of intervention impact. Moreover, this study involved multiple statistical tests across several outcomes and timepoints, increasing the risk of Type I error inflation. As this was a pilot study with an exploratory aim, formal correction for multiple comparisons was not applied. Consequently, findings should be interpreted cautiously and considered preliminary, rather than confirmatory. A further limitation is that the inclusion criterion of PGSI ≥ 3 resulted in a sample with predominantly moderate gambling severity. While appropriate for an early‐intervention model such as SBIRT, this may have limited the magnitude of observable intervention effects and restricts generalisability to individuals with more severe gambling problems. Finally, gambling frequency and expenditure were assessed using single open‐ended self‐report items, which may be subject to recall bias and imprecision. Future research would benefit from using more structured, activity‐specific measures to improve the reliability and interpretability of gambling behaviour outcomes.

## 5 Conclusion

This randomised online pilot trial evaluated a brief, digitally delivered adaptation of SBIRT components for gambling‐related harm. Although improvements were observed over time across both conditions, between‐condition differences were limited, suggesting that the intervention did not produce consistent benefits beyond those associated with assessment and information provision. Future research should focus on refining intervention components and evaluating SBIRT interventions in contexts which are more conducive to behaviour change.

## Author Contributions

All authors contributed to the conception and design, analysis, interpretation of the results, writing, and revision of the manuscript.

## Ethics Statement

Ethical approval was obtained from Swansea University Human Research Ethics Committee (1 2025 11481 11793).

## Conflicts of Interest

The authors declare no conflicts of interest.

## Supporting information


Supporting File

## Data Availability

The data that support the findings of this study are openly available in Open Science Framework at https://osf.io/cq5j9/overview?view_only=bafe1fb359c048c7abea1bd7e5a397da.
