# Supplementary material for: Evaluating the Efficacy of Electronic Screening, Brief Intervention, and Referral to Treatment (e‐SBIRT) for Gambling: An Online Pilot Randomised Trial
Source: J Clin Psychol. 2026 Jul 10;82(10):1628–38. doi: 10.1002/jclp.70188 (PMC13544470; doi:10.1002/jclp.70188)
Supplement: Supplementary file 1 [file JCLP-82-1628-s001.docx]

**Supplementary Materials**

**Supplementary Material 1.1 – e-SBIRT brief intervention: Additional Information**

Normative feedback on gambling: Normative feedback was provided based on the participants PGSI score and age. If participants scored in the non-problem or low-risk categories, they received age-based normative feedback with an affirming message and viewed tips on how to maintain safe gambling before being directed out of the survey. If participants scored in the moderate or high-risk categories, they received age-based normative feedback, a supportive message and were directed to the next section of the e-SBIRT. Data used in the normative feedback was taken from the Gambling Survey for Great Britain (Wardle et al., 2023).

Psychoeducation on the consequences of gambling: Participants were provided with a statement detailing the frequency of individuals that have experienced negative consequences due to their gambling in the past year. Participants were then presented an infographic of the common negative consequences arising from gambling. Data used in this section was taken from the Gambling Survey for Great Britain (Wardle et al., 2023).

Assessment of readiness to change with feedback: Readiness to change was assessed using the Gambling Readiness to Change Questionnaire (GRTC) (Neighbors, 2002). The GRTC is adapted from the Alcohol Readiness To Change Questionnaire ([Rollnick et al., 1992](https://www.ncbi.nlm.nih.gov/pmc/articles/PMC1797803/#R30)) and is a 9-item scale with three items measuring each of the three stages: precontemplation, contemplation and action. In this, participants indicate the extent to which they agree with each item, from 1 (strongly disagree) to 5 (strongly agree). The GRTC has demonstrated satisfactory reliability for the composite scale (alpha = 0.81) and each of the three subscales, with alphas of 0.64, 0.80 and 0.74 for precontemplation, contemplation and action respectively (Neighbors, 2002). After completion of the GRTC, participants received feedback on their current stage of change alongside an affirming message. Consistent with Miller and Rollnick (1991), participants that asserted they were in the pre-contemplation or contemplation stage were directed to a decisional balance exercise designed to increase motivation for behaviour change. Participants that asserted they were in the action stage were directed to a goal setting exercise, as they had already acknowledged a need for behaviour change (Neighbors, 2002).

Decisional balance exercise: Participants were informed the exercise was designed to help with their motivation for change by reviewing their personal relationship with gambling. Participants were then instructed to write up to three positive and negative things about their gambling, before ranking them in terms of how important they are to them. Participants were encouraged to reflect on their motivation for change having completed the exercise.

Goal setting exercise: This exercise was adapted from the HERA exercise ‘Stairway to Change - where are you?’ (Boudreaux et al., 2013). Participants viewed an image with some example goals relating to gambling and were instructed to reflect on their own goals. Participants then completed up to three of their own goals before completing fields on how and when they were going to complete these.

Relapse prevention exercise: This exercise was adapted from the HERA exercise ‘Breaking the chains’ (Boudreaux et al., 2013). In this, participants were provided with information on gambling triggers and viewed an image with some example triggers. They were then instructed to write up to five of their own triggers and then write a plan on how to manage these without engaging in gambling.

**Supplementary Material 1.2 – Linear mixed effect models: Main effects of condition, time and condition x time interaction**

| **Gambling harms  (SGHS)** | Numerator df | Denominator df | *F* | Sig. |
| --- | --- | --- | --- | --- |
| Condition | 1 | 81.794 | .521 | .472 |
| Time | 2 | 151.133 | 4.135 | **.018** |
| Condition * Time | 2 | 151.133 | .148 | .863 |
| **Gambling frequency (hours)** |  |  |  |  |
| Condition | 1 | 76.46 | 1.426 | .236 |
| Time | 2 | 147.068 | 2.015 | .137 |
| Condition * Time | 2 | 147.068 | .590 | .556 |
| **Gambling expenditure (£)** |  |  |  |  |
| Condition | 1 | 84.861 | .465 | .497 |
| Time | 2 | 160.166 | .939 | .393 |
| Condition * Time | 2 | 160.166 | 1.235 | .294 |
| **Gambling symptom severity (G-SAS)** |  |  |  |  |
| Condition | 1 | 81.760 | .262 | .610 |
| Time | 2 | 151.385 | .871 | .421 |
| Condition * Time | 2 | 151.385 | .586 | .558 |
| **Perceived ability to control gambling (0 – 10)** |  |  |  |  |
| Condition | 1 | 81.559 | .826 | .366 |
| Time | 2 | 151.260 | 3.479 | **.033** |
| Condition * Time | 2 | 151.260 | 2.706 | .070 |
| **Quality of life (EUROHIS - QOL-8)** |  |  |  |  |
| Condition | 1 | 81.123 | .156 | .694 |
| Time | 2 | 149.841 | .181 | .835 |
| Condition * Time | 2 | 149.841 | 1.946 | .146 |

**Supplementary Material 1.3 – Linear mixed effect models: Pairwise comparisons for condition and time**

| **Gambling harms (SGHS) - Condition** | | | | | | | |
| --- | --- | --- | --- | --- | --- | --- | --- |
| (I) Group | (J) Group | Mean Difference (I-J) | Std. Error | df | Sig.^b^ | 95% Confidence Interval for Difference^b^ | |
|  |  |  |  |  |  | Lower Bound | Upper Bound |
| intervention | control | .439 | .608 | 81.794 | .472 | -.770 | 1.648 |
| control | intervention | -.439 | .608 | 81.794 | .472 | -1.648 | .770 |
| Based on estimated marginal means | | | | | | | |
| a. Dependent Variable: harms_all_time. | | | | | | | |
| b. Adjustment for multiple comparisons: Bonferroni. | | | | | | | |

| **Gambling harms (SGHS) – Time** | | | | | | | |
| --- | --- | --- | --- | --- | --- | --- | --- |
| (I) Time | (J) Time | Mean Difference (I-J) | Std. Error | df | Sig.^c^ | 95% Confidence Interval for Difference^c^ | |
|  |  |  |  |  |  | Lower Bound | Upper Bound |
| 0 weeks | 4 weeks | .583^*^ | .227 | 151.185 | .033 | .034 | 1.131 |
|  | 12 weeks | .544 | .230 | 151.501 | .058 | -.013 | 1.100 |
| 4 weeks | 0 weeks | -.583^*^ | .227 | 151.185 | .033 | -1.131 | -.034 |
|  | 12 weeks | -.039 | .232 | 150.701 | 1.000 | -.601 | .524 |
| 12 weeks | 0 weeks | -.544 | .230 | 151.501 | .058 | -1.100 | .013 |
|  | 4 weeks | .039 | .232 | 150.701 | 1.000 | -.524 | .601 |
| Based on estimated marginal means | | | | | | | |
| *. The mean difference is significant at the .05 level. | | | | | | | |
| a. Dependent Variable: harms_all_time. | | | | | | | |
| c. Adjustment for multiple comparisons: Bonferroni. | | | | | | | |

| **Gambling frequency (hours) - Condition** | | | | | | | |
| --- | --- | --- | --- | --- | --- | --- | --- |
| (I) Group | (J) Group | Mean Difference (I-J) | Std. Error | df | Sig.^b^ | 95% Confidence Interval for Difference^b^ | |
|  |  |  |  |  |  | Lower Bound | Upper Bound |
| intervention | control | 5.348 | 4.479 | 76.463 | .236 | -3.572 | 14.268 |
| control | intervention | -5.348 | 4.479 | 76.463 | .236 | -14.268 | 3.572 |
| Based on estimated marginal means | | | | | | | |
| a. Dependent Variable: Frequency_LongForm. | | | | | | | |
| b. Adjustment for multiple comparisons: Bonferroni. | | | | | | | |

| **Gambling frequency (hours) - Time** | | | | | | | |
| --- | --- | --- | --- | --- | --- | --- | --- |
| (I) Time | (J) Time | Mean Difference (I-J) | Std. Error | df | Sig.^b^ | 95% Confidence Interval for Difference^b^ | |
|  |  |  |  |  |  | Lower Bound | Upper Bound |
| 0 weeks | 4 weeks | 2.604 | 2.431 | 147.027 | .857 | -3.282 | 8.490 |
|  | 12 weeks | 4.936 | 2.463 | 147.688 | .141 | -1.029 | 10.901 |
| 4 weeks | 0 weeks | -2.604 | 2.431 | 147.027 | .857 | -8.490 | 3.282 |
|  | 12 weeks | 2.332 | 2.495 | 146.475 | 1.000 | -3.709 | 8.373 |
| 12 weeks | 0 weeks | -4.936 | 2.463 | 147.688 | .141 | -10.901 | 1.029 |
|  | 4 weeks | -2.332 | 2.495 | 146.475 | 1.000 | -8.373 | 3.709 |
| Based on estimated marginal means | | | | | | | |
| a. Dependent Variable: Frequency_LongForm. | | | | | | | |
| b. Adjustment for multiple comparisons: Bonferroni. | | | | | | | |

| **Gambling expenditure (£) - Condition** | | | | | | | |
| --- | --- | --- | --- | --- | --- | --- | --- |
| (I) Group | (J) Group | Mean Difference (I-J) | Std. Error | df | Sig.^b^ | 95% Confidence Interval for Difference^b^ | |
|  |  |  |  |  |  | Lower Bound | Upper Bound |
| intervention | control | 34.598 | 50.765 | 84.861 | .497 | -66.338 | 135.535 |
| control | intervention | -34.598 | 50.765 | 84.861 | .497 | -135.535 | 66.338 |
| Based on estimated marginal means | | | | | | | |
| a. Dependent Variable: Expenditure_LongForm. | | | | | | | |
| b. Adjustment for multiple comparisons: Bonferroni. | | | | | | | |

| **Gambling expenditure (£) - Time** | | | | | | | |
| --- | --- | --- | --- | --- | --- | --- | --- |
| (I) Time | (J) Time | Mean Difference (I-J) | Std. Error | df | Sig.^b^ | 95% Confidence Interval for Difference^b^ | |
|  |  |  |  |  |  | Lower Bound | Upper Bound |
| 0 weeks | 4 weeks | -42.425 | 31.335 | 160.037 | .533 | -118.238 | 33.388 |
|  | 12 weeks | -26.546 | 31.747 | 160.889 | 1.000 | -103.351 | 50.258 |
| 4 weeks | 0 weeks | 42.425 | 31.335 | 160.037 | .533 | -33.388 | 118.238 |
|  | 12 weeks | 15.879 | 32.166 | 159.559 | 1.000 | -61.947 | 93.705 |
| 12 weeks | 0 weeks | 26.546 | 31.747 | 160.889 | 1.000 | -50.258 | 103.351 |
|  | 4 weeks | -15.879 | 32.166 | 159.559 | 1.000 | -93.705 | 61.947 |
| Based on estimated marginal means | | | | | | | |
| a. Dependent Variable: Expenditure_LongForm. | | | | | | | |
| b. Adjustment for multiple comparisons: Bonferroni. | | | | | | | |

| **Gambling symptom severity (G-SAS) – Condition** | | | | | | | |
| --- | --- | --- | --- | --- | --- | --- | --- |
| (I) Group | (J) Group | Mean Difference (I-J) | Std. Error | df | Sig.^b^ | 95% Confidence Interval for Difference^b^ | |
|  |  |  |  |  |  | Lower Bound | Upper Bound |
| intervention | control | -.914 | 1.787 | 81.760 | .610 | -4.468 | 2.640 |
| control | intervention | .914 | 1.787 | 81.760 | .610 | -2.640 | 4.468 |
| Based on estimated marginal means | | | | | | | |
| a. Dependent Variable: gsas_all_time. | | | | | | | |
| b. Adjustment for multiple comparisons: Bonferroni. | | | | | | | |

| **Gambling symptom severity (G-SAS) – Time** | | | | | | | |
| --- | --- | --- | --- | --- | --- | --- | --- |
| (I) Time | (J) Time | Mean Difference (I-J) | Std. Error | df | Sig.^b^ | 95% Confidence Interval for Difference^b^ | |
|  |  |  |  |  |  | Lower Bound | Upper Bound |
| 0 weeks | 4 weeks | .363 | .739 | 151.427 | 1.000 | -1.427 | 2.153 |
|  | 12 weeks | .982 | .750 | 151.813 | .577 | -.833 | 2.797 |
| 4 weeks | 0 weeks | -.363 | .739 | 151.427 | 1.000 | -2.153 | 1.427 |
|  | 12 weeks | .619 | .758 | 150.902 | 1.000 | -1.217 | 2.455 |
| 12 weeks | 0 weeks | -.982 | .750 | 151.813 | .577 | -2.797 | .833 |
|  | 4 weeks | -.619 | .758 | 150.902 | 1.000 | -2.455 | 1.217 |
| Based on estimated marginal means | | | | | | | |
| a. Dependent Variable: gsas_all_time. | | | | | | | |
| b. Adjustment for multiple comparisons: Bonferroni. | | | | | | | |

| **Perceived ability to control gambling (0 - 10) – Condition** | | | | | | | |
| --- | --- | --- | --- | --- | --- | --- | --- |
| (I) Group | (J) Group | Mean Difference (I-J) | Std. Error | df | Sig.^b^ | 95% Confidence Interval for Difference^b^ | |
|  |  |  |  |  |  | Lower Bound | Upper Bound |
| intervention | control | -.408 | .448 | 81.559 | .366 | -1.300 | .484 |
| control | intervention | .408 | .448 | 81.559 | .366 | -.484 | 1.300 |
| Based on estimated marginal means | | | | | | | |
| a. Dependent Variable: selfefficacy_all_time. | | | | | | | |
| b. Adjustment for multiple comparisons: Bonferroni. | | | | | | | |

| **Perceived ability to control gambling (0 – 10) – Time** | | | | | | | |
| --- | --- | --- | --- | --- | --- | --- | --- |
| (I) Time | (J) Time | Mean Difference (I-J) | Std. Error | df | Sig.^c^ | 95% Confidence Interval for Difference^c^ | |
|  |  |  |  |  |  | Lower Bound | Upper Bound |
| 0 weeks | 4 weeks | -.287 | .190 | 151.299 | .397 | -.747 | .172 |
|  | 12 weeks | -.505^*^ | .193 | 151.703 | .029 | -.971 | -.039 |
| 4 weeks | 0 weeks | .287 | .190 | 151.299 | .397 | -.172 | .747 |
|  | 12 weeks | -.218 | .195 | 150.765 | .797 | -.690 | .254 |
| 12 weeks | 0 weeks | .505^*^ | .193 | 151.703 | .029 | .039 | .971 |
|  | 4 weeks | .218 | .195 | 150.765 | .797 | -.254 | .690 |
| Based on estimated marginal means | | | | | | | |
| *. The mean difference is significant at the .05 level. | | | | | | | |
| a. Dependent Variable: selfefficacy_all_time. | | | | | | | |
| c. Adjustment for multiple comparisons: Bonferroni. | | | | | | | |

| **Quality of life (EUROHIS - QOL-8) – Condition** | | | | | | | |
| --- | --- | --- | --- | --- | --- | --- | --- |
| (I) Group | (J) Group | Mean Difference (I-J) | Std. Error | df | Sig.^b^ | 95% Confidence Interval for Difference^b^ | |
|  |  |  |  |  |  | Lower Bound | Upper Bound |
| intervention | control | .512 | 1.295 | 81.123 | .694 | -2.065 | 3.090 |
| control | intervention | -.512 | 1.295 | 81.123 | .694 | -3.090 | 2.065 |
| Based on estimated marginal means | | | | | | | |
| a. Dependent Variable: qol_all_time. | | | | | | | |
| b. Adjustment for multiple comparisons: Bonferroni. | | | | | | | |

| **Quality of life (EUROHIS - QOL-8) – Time** | | | | | | | |
| --- | --- | --- | --- | --- | --- | --- | --- |
| (I) Time | (J) Time | Mean Difference (I-J) | Std. Error | df | Sig.^b^ | 95% Confidence Interval for Difference^b^ | |
|  |  |  |  |  |  | Lower Bound | Upper Bound |
| 0 weeks | 4 weeks | .082 | .337 | 149.890 | 1.000 | -.735 | .898 |
|  | 12 weeks | -.125 | .342 | 150.047 | 1.000 | -.953 | .703 |
| 4 weeks | 0 weeks | -.082 | .337 | 149.890 | 1.000 | -.898 | .735 |
|  | 12 weeks | -.207 | .346 | 149.579 | 1.000 | -1.044 | .630 |
| 12 weeks | 0 weeks | .125 | .342 | 150.047 | 1.000 | -.703 | .953 |
|  | 4 weeks | .207 | .346 | 149.579 | 1.000 | -.630 | 1.044 |
| Based on estimated marginal means | | | | | | | |
| a. Dependent Variable: qol_all_time. | | | | | | | |
| b. Adjustment for multiple comparisons: Bonferroni. | | | | | | | |

**Supplementary Material 1.4 – Linear mixed effect models: Estimated marginal means for time and condition of each variable**

| **Gambling harms (SGHS)** | | | | | | |
| --- | --- | --- | --- | --- | --- | --- |
| Group | Time | Mean | Std. Error | df | 95% Confidence Interval | |
|  |  |  |  |  | Lower Bound | Upper Bound |
| intervention | 0 weeks | 4.214 | .461 | 109.519 | 3.300 | 5.128 |
|  | 4 weeks | 3.592 | .463 | 110.983 | 2.674 | 4.509 |
|  | 12 weeks | 3.755 | .467 | 114.124 | 2.830 | 4.680 |
| control | 0 weeks | 3.805 | .467 | 109.519 | 2.880 | 4.730 |
|  | 4 weeks | 3.262 | .476 | 116.592 | 2.319 | 4.206 |
|  | 12 weeks | 3.177 | .479 | 118.411 | 2.230 | 4.125 |
| a. Dependent Variable: harms_all_time. | | | | | | |

| **Gambling frequency (hours)** | | | | | | |
| --- | --- | --- | --- | --- | --- | --- |
| Group | Time | Mean | Std. Error | df | 95% Confidence Interval | |
|  |  |  |  |  | Lower Bound | Upper Bound |
| intervention | 0 weeks | 18.976 | 3.665 | 135.033 | 11.729 | 26.224 |
|  | 4 weeks | 18.941 | 3.690 | 137.614 | 11.645 | 26.237 |
|  | 12 weeks | 15.834 | 3.744 | 143.057 | 8.434 | 23.234 |
| control | 0 weeks | 16.537 | 3.709 | 135.033 | 9.201 | 23.872 |
|  | 4 weeks | 11.364 | 3.834 | 145.906 | 3.787 | 18.941 |
|  | 12 weeks | 9.807 | 3.865 | 148.937 | 2.170 | 17.444 |
| a. Dependent Variable: Frequency_LongForm. | | | | | | |

| **Gambling expenditure (£)** | | | | | | |
| --- | --- | --- | --- | --- | --- | --- |
| Time | Time | Mean | Std. Error | df | 95% Confidence Interval | |
|  |  |  |  |  | Lower Bound | Upper Bound |
| intervention | 0 weeks | 178.905 | 43.137 | 163.711 | 93.728 | 264.081 |
|  | 4 weeks | 265.029 | 43.485 | 166.625 | 179.177 | 350.881 |
|  | 12 weeks | 206.512 | 44.225 | 172.656 | 119.221 | 293.803 |
| control | 0 weeks | 174.146 | 43.660 | 163.711 | 87.938 | 260.355 |
|  | 4 weeks | 172.872 | 45.338 | 175.114 | 83.392 | 262.352 |
|  | 12 weeks | 172.872 | 45.766 | 178.369 | 109.319 | 289.944 |
| a. Dependent Variable: Expenditure_LongForm. | | | | | | |

| **Gambling symptom severity (G-SAS)** | | | | | | |
| --- | --- | --- | --- | --- | --- | --- |
| Group | Time | Mean | Std. Error | df | 95% Confidence Interval | |
|  |  |  |  |  | Lower Bound | Upper Bound |
| intervention | 0 weeks | 17.810 | 1.378 | 116.071 | 15.080 | 20.539 |
|  | 4 weeks | 17.099 | 1.385 | 117.819 | 14.357 | 19.841 |
|  | 12 weeks | 16.017 | 1.398 | 121.560 | 13.249 | 18.786 |
| control | 0 weeks | 17.951 | 1.395 | 116.071 | 15.188 | 20.714 |
|  | 4 weeks | 17.936 | 1.428 | 124.268 | 15.110 | 20.763 |
|  | 12 weeks | 17.780 | 1.436 | 126.417 | 14.938 | 20.622 |
| a. Dependent Variable: gsas_all_time. | | | | | | |

| **Perceived ability to control gambling (0-10)** | | | | | | |
| --- | --- | --- | --- | --- | --- | --- |
| Group | Time | Mean | Std. Error | df | 95% Confidence Interval | |
|  |  |  |  |  | Lower Bound | Upper Bound |
| intervention | 0 weeks | 6.452 | .347 | 117.547 | 5.765 | 7.140 |
|  | 4 weeks | 6.740 | .349 | 119.364 | 6.049 | 7.431 |
|  | 12 weeks | 7.350 | .353 | 123.249 | 6.652 | 8.048 |
| control | 0 weeks | 7.122 | .352 | 117.547 | 6.426 | 7.818 |
|  | 4 weeks | 7.409 | .360 | 126.002 | 6.697 | 8.122 |
|  | 12 weeks | 7.234 | .362 | 128.228 | 6.517 | 7.951 |
| a. Dependent Variable: selfefficacy_all_time. | | | | | | |

| **Quality of life (EUROHIS - QOL-8)** | | | | | | |
| --- | --- | --- | --- | --- | --- | --- |
| Group | Time | Mean | Std. Error | df | 95% Confidence Interval | |
|  |  |  |  |  | Lower Bound | Upper Bound |
| intervention | 0 weeks | 27.548 | .946 | 94.396 | 25.669 | 29.427 |
|  | 4 weeks | 28.067 | .948 | 95.143 | 26.184 | 29.950 |
|  | 12 weeks | 27.713 | .953 | 96.753 | 25.822 | 29.604 |
| control | 0 weeks | 27.463 | .958 | 94.396 | 25.562 | 29.365 |
|  | 4 weeks | 26.780 | .969 | 98.259 | 24.858 | 28.703 |
|  | 12 weeks | 27.548 | .971 | 99.203 | 25.621 | 29.475 |
| a. Dependent Variable: qol_all_time. | | | | | | |
